# L’hypothèse génétique de la maladie de Haglund, et son association avec la spondyloarthrite ankylosante: à propos d’un cas

**DOI:** 10.11604/pamj.2021.38.49.27541

**Published:** 2021-01-18

**Authors:** Hicham Douma, Abdelkrim El Hassani, Faycal Rifki, Ouahb Azriouil, Basma Dihi, Houda El Madkouri, Laila Liqali, Khalid Koulali Idrissi

**Affiliations:** 1 Department of Traumatology, Avicenne Military Hospital, Faculty of Medicine and Pharmacy of Marrakech, University Cadi Ayyad of Marrakech, Marrakech, Morocco,; 2 Department of Rheumatology, Avicenne Military Hospital, Faculty of Medicine and Pharmacy of Marrakech, University Cadi Ayyad of Marrakech, Marrakech, Morocco,; 3 Department of Radiology, Avicenne Military Hospital, Faculty of Medicine and Pharmacy of Marrakech, University Cadi Ayyad of Marrakech, Marrakech, Morocco

**Keywords:** Haglund, spondyloarthrite ankylosante, resection, talalgie, *case report*, Haglund, ankylosing spondylitis, resection, talalgia, case report

## Abstract

La maladie de Haglund est une pathologie caractérisée par une excroissance osseuse postéro-supérieure du calcanéum, induisant un conflit avec le tendon d´Achille, et se manifestant principalement par une talalgie postérieure. Nous rapportons l´observation d´une jeune femme de 28 ans suivie pour spondylarthrite ankylosante, avec une maman suivie pour la même maladie systémique, et une tante maternelle opérée pour maladie de Haglund. La patiente a trop trainée pour avoir le diagnostic vu que la talalgie a été considérée comme enthésite de sa maladie de système. La patiente ne s´est pas améliorée sous traitement médical. La chirurgie nous a donné des résultats satisfaisants. Ce qui soulève les hypothèses du caractère génétique et héréditaire du syndrome de Haglund, et sa relation avec la réponse au traitement médical.

## Abstract (en)

Haglund´s syndrome is characterized by osterosuperior calcaneal exostosis which irritates the Achilles tendon and manifests mainly as posterior talalgia. We here report the case of a 28-year-old woman on follow up for ankylosing spondylitis. Her mother was on follow up for the same systemic disease, and one of her maternal aunts had undergone surgery due to Haglund’s syndrome. Patient’s delay led to incorrect diagnosis as talagia was considered by physicians as a consequence of her system disease. The patient did not improve under medical treatment. Surgery outcome was satisfactory. This study raises a hypothesis about the genetic and hereditary character of Haglund’s syndrome and its relationship with treatment response.

## Introduction

La maladie de Haglund est définie par l´existence d´une excroissance osseuse au niveau de l´angle postéro-supérieur du calcanéus, engendrant une inflammation du tendon d´Achille qui peut aller jusqu'à sa rupture. Nous présentons un cas clinique rare d´une patiente suivie pour spondylarthrite ankylosante, avec un antécédent familial de la maladie de Haglund.

## Patient et observation

**Figure 1:**
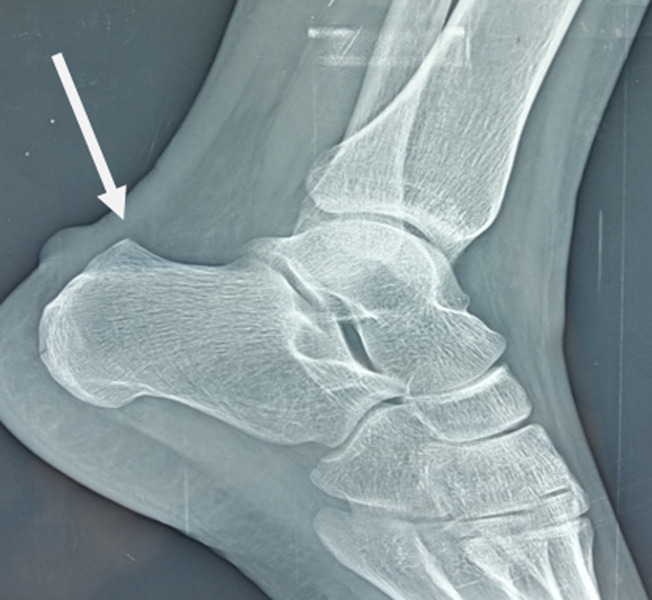
radiographie de profil du pied droit montrant la maladie de Haglund

Mme N.L, âgée de 28 ans, suivie pour spondylarthrite ankylosante depuis 3 ans, et ayant comme antécédents familiaux une tante maternelle opérée en 2008 pour maladie de Haglund bilatérale, et une maman atteinte de spondylarthrite ankylosante (SPA). Elle s´est présentée à la consultation pour aggravation des talalgies postérieures droites depuis 4 mois. La patiente déclare la persistance de la douleur malgré le fait qu´elle ne soit pas en poussée de sa maladie systémique depuis 3 mois. La patiente déclare l´échec des anti-inflammatoires et de l´infiltration cortisonique a une dose de 50 mg en péri tendineux précédemment faite. La patiente a déjà été mise par son rhumatologue sous traitement de fond par Salazopyrine puis les anti-TNF alpha, sans amélioration de ses douleurs de l´arrière-pied. L´examen clinique a mis en évidence une tuméfaction douloureuse à la palpation rétro calcanéenne. Le signe de Thompson était négatif, avec stabilité de la cheville conservée. Une radiographie du pied droit de face et de profil ont été demandées (Figure 1). Une excroissance osseuse retro-calcanéenne était bien visible sur la radio de profil.

**Figure 2:**
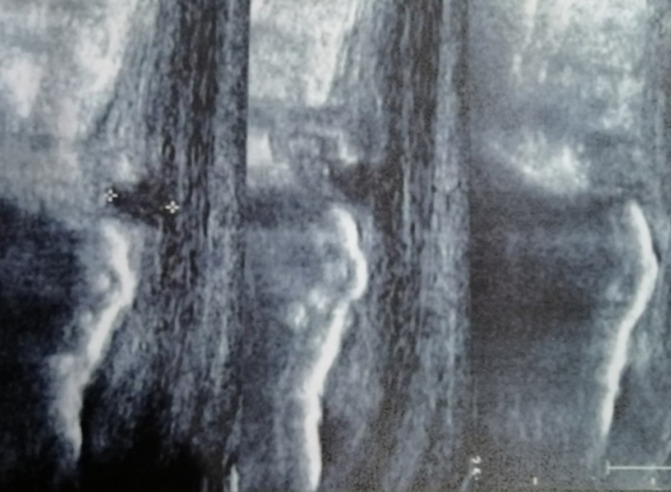
échographie montrant une tendinite achilléenne avec bursite

**Figure 3:**
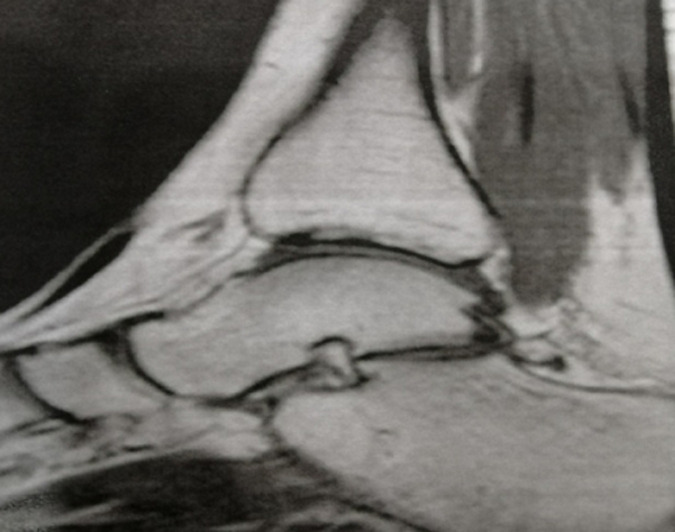
IRM montrant la tendinite achilléenne avec bursite pré-achilléenne

**Figure 4:**
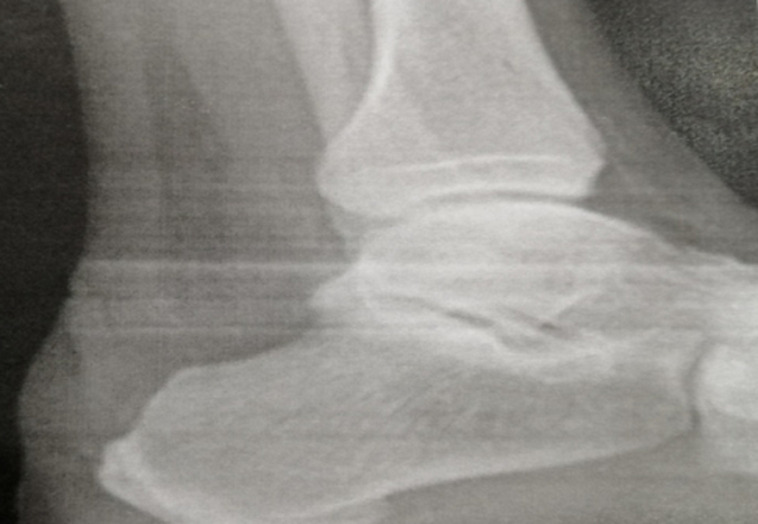
image radiographique par amplificateur de brillance après résection de l´exostose osseuse du calcanéum

Le diagnostic de la maladie de Haglund a été retenu. L´angle de Chauveaux était à 17 degrés. L´angle de Fowler-Philip était à 83°C. Vu l´échec du traitement médical, la patiente a été traitée par résection chirurgicale par voie conventionnelle de l´exostose associée à une excision de la bourse retro calcanéenne. La voie d´abord était latérale para-achilienne en J. Une mise en décharge pendant 6 semaines avec immobilisation de la cheville en équin par résine, suivie par rééducation de la cheville droite ont été indiquées. Après un recul de 6 mois, l´évolution a été largement satisfaisante, marquée par une disparition des douleurs avec reprise de la mobilité de la cheville avec des bonnes amplitudes. L´antécédent familial de la maladie de Haglund et sa résistance au traitement médical dans notre cas soulèvent deux grandes hypothèses, qui sont le rôle de la génétique dans l´apparition et la résistance de la maladie de Haglund au traitement médical, et sa relation avec d´autres maladies de système (Figure 2, Figure 3, Figure 4).

## Discussion

Le syndrome de Haglund ou syndrome du calcanéum bossu, a été décrit la première fois par un suédois Patrick Haglund en 1928 [1]; qui est une cause de douleurs mécaniques de l´arrière pied à type de talalgie postérieure handicapante, suite à l´existence d´un conflit calcanéo-achilléen due à une exostose mesurant environ 5-20mm, pointue et saillante en haut et en arrière de la grosse tubérosité du calcanéum, engendrant une inflammation du tendon d´Achille et de la bourse rétro-calcanéenne et rétro-achilléenne [2]. C´est une pathologie généralement du sujet jeune entre 20-40 ans, le plus souvent bilatéral [3]. Elle prédomine chez le sexe féminin [4]. Bien que sa pathogénie soit inconnue, certains facteurs mécaniques prédisposent à ce syndrome tel que: les chaussures trop serrées, les talons hauts portés par les femmes, un pied creux qui met en tension le tendon ce qui augmente la friction entre ce dernier et l´os [2].

Plusieurs étiopathogénies ont été évoquées afin d´expliquer ce syndrome [5]; dysplasie: corrélation entre la clinique et les images radiologiques; rhumatismale: rarement il est associé à des maladies rhumatismales: la goutte, la spondylarthrite ankylosante (SPA) (c´est le cas de notre patiente); traumatique: les microtraumatismes entre le triceps sural et le calcanéum induisent une inflammation de la bourse retro-calcanéenne puis une ossification réactionnelle.

Certains auteurs déclarent une hypothèse de prédisposition génétique. Le tableau clinique se caractérise par une douleur mécanique de l´arrière pied accentuée par l´activité physique, chaussage serrée et la flexion dorsale et plantaire de la cheville et qui répond le plus souvent aux anti-inflammatoires, cela peut solliciter autres diagnostiques qu´il faut y penser avant de retenir la maladie de Haglund tel que: la goutte; SPA; une fracture de fatigue de calcanéum. A l´examen clinique on trouve une tuméfaction douloureuse et rouge du talon, qui peut rarement évoluer vers une ulcération du talon, tandis que la stabilité de la cheville est conservée et signe de Thompson est négatif [3].

Sur le plan radiologique, la radiographie standard suffit surtout l´incidence de profil pour poser le diagnostic et permet de visualiser la proéminence et mesurer certains angles notamment l´angle de Chauveaux qui est plus utile pour indiquer la chirurgie, il correspond à la différence entre l´angle de verticalisation du calcanéum a (tangente de bord inferieur du calcanéum et horizontal) et l´angle postérieur du calcanéum ß (tangente du bord postérieur du calcanéum perpendiculaire au sol et la ligne unissant le point du contact de cette tangente et le sommet de la grosse tubérosité). L´angle de Chauveaux est normalement 10°C. Il y´a d´autres angles moins utilisés comme angle de Fowler et Philip entre 60°-75°C [1]. Autres signes indirects: déformation ou perte du triangle de Kager, limité en avant par le tendon du long fléchisseur de l´hallux, en arrière par le tendon d´Achille, et en bas par le calcanéum [6].

L´échographie n´est pas indispensable pour le diagnostic. Elle met en évidence l´épaississement inflammatoire du tendon d´Achille et la bursite [7]. L´imagerie par résonance magnétique (IRM) est demandée si le diagnostic est incertain. L´excroissance osseuse est bien visualisée en séquence T1. Elle permet aussi de faire un bilan lésionnel détaillé du tendon achilléen (anomalie d´insertion, déchirure, épaississement, rupture) [8].

Sur le plan thérapeutique, le traitement conservateur (médical et physique) est systématique et est le premier à instaurer, il permet la guérison dans 90% des cas [7]. Il soulage la douleur, il diminue l´inflammation et protège le tendon; Traitement médical: AINS général ou local, infiltration des corticoïdes en péri-tendineux et jamais en intra-tendineux, car risque de rupture du tendon déjà en conflit mécanique; Traitement physique: le repos et l´hygiène de vie. La rééducation à une grande place, et elle consiste en un massage transversal du tendon et étirement des chaines postérieures; Traitement orthopédique consiste à adapter les chaussures et la correction des troubles statiques; La cryothérapie et l´utilisation des ultrasons peuvent être utiles aussi [1].

En cas d´échec, on a recourt à la chirurgie, qu´on peut faire par voie conventionnelle ou arthroscopique. La résection de cette exostose est la règle d´or, associée au débridement du tissu inflammatoire et à une excision de la bourse. Si le tendon est lésé, on fait un peignage de ce dernier pour éviter une réintervention. Zadek et Taylor en 1939 ont proposé une ostéotomie à base supérieure qui a pour but d´avancer l´angle postéro-supérieur du calcanéum afin de diminuer le conflit avec la face antérieure du tendon [5].

Le choix entre ces deux techniques dépend du type d´anomalie architecturale, l´importance de la déformation et la demande du patient [1]. Après l´intervention, on immobilise la cheville par la résine en équin sans appui pendant 6 semaines suivie par une rééducation avec anticoagulant à 0,4cc /j.

Les complications de cette chirurgie sont rares 2%: infection; algodystrophie; rupture ou désinsertion du tendon due à une ostéotomie massive; lésion de nerf sural ou tibial postérieur. Notre cas est original et pertinent, car il met le doigt sur une hypothèse étiopathogénique extrêmement rare, qui est le terrain génétique de cette pathologie, et le rôle de l´hérédité dans l´apparition et la transmission de la maladie de Haglund, et de sa résistance au traitement médical.

## Conclusion

La maladie de Haglund doit être toujours suspectée devant des talalgies uni ou bilatérale, avec ou sans antécédent de maladie de système. Une radio du pied de profil est généralement suffisante pour poser le diagnostic. La maladie répond le plus souvent au traitement médical. La chirurgie est envisagée en cas d´échec de celui-ci. Notre cas clinique met en évidence le caractère génétique de la maladie et sa relation avec la réponse de ce syndrome au traitement médical.
